# Differential efficacy of segmentectomy and wedge resection in sublobar resection compared to lobectomy for solid-dominant stage IA lung cancer: a systematic review and meta-analysis

**DOI:** 10.1097/JS9.0000000000000896

**Published:** 2023-11-16

**Authors:** Huahang Lin, Zhiyu Peng, Ke Zhou, Linchuan Liang, Jie Cao, Zhaokang Huang, Lonqi Chen, Jiandong Mei

**Affiliations:** a Department of Thoracic Surgery, West China Hospital; b Western China Collaborative Innovation Center for Early Diagnosis and Multidisciplinary Therapy of Lung Cancer, Sichuan University, Chengdu, Sichuan Province, People’s Republic of China

**Keywords:** lobectomy, lung cancer, solid-dominant, stage IA, sublobar resection

## Abstract

**Background:**

Currently, the impact of sublobar resection versus lobectomy on the prognosis of solid-dominant stage IA lung cancer is contradictory in different studies, which requires further exploration.

**Methods:**

The authors analyzed 26 studies, including one randomized controlled trial and retrospective cohort studies. Pooled hazard ratios (HRs) and 95% confidence intervals (CIs) were calculated using fixed-effects or random-effects models based on heterogeneity levels.

**Results:**

The analysis included 12 667 patients, with 3488 undergoing sublobar resections and 9179 receiving lobectomies. The overall analysis revealed no statistically significant difference in overall survival (OS) (HR=1.28, 95% CI: 0.98–1.69) between sublobar resection and lobectomy, but lobectomy was associated with better recurrence-free survival (RFS) (HR=1.39, 95% CI: 1.10–1.75). Subgroup analyses revealed that, for tumors with a diameter ≤2 cm, sublobar resection versus lobectomy showed no significant difference in OS but sublobar resection had lower RFS. For 2–3 cm tumors, both OS and RFS were significantly lower in the sublobar resection group. When consolidation-to-tumor ratio (CTR) ranged from 0.5 to <1, OS did not differ significantly, but RFS was significantly lower in sublobar resection. Lung cancers with CTR=1 showed significantly lower OS and RFS in the sublobar resection group. Segmentectomy provided similar OS and RFS compared to lobectomy, while wedge resection had a detrimental effect on patient prognosis. However, wedge resection may have provided comparable outcomes for patients aged 75 years or older.

**Conclusion:**

Our findings suggest that segmentectomy and lobectomy yield similar oncological outcomes. However, compared to lobectomy, wedge resection is associated with a poorer prognosis. Nevertheless, for elderly patients, wedge resection is also a reasonable surgical option.

## Introduction

Highlights

- Lung cancer is a complex disease with different subtypes, requiring a personalized approach to management.
- Sublobar resection, such as segmentectomy and wedge resection, has emerged as an alternative surgical approach to preserve lung function while ensuring oncologic control.
- The meta-analysis revealed that segmentectomy exhibited comparable overall survival (OS) and recurrence-free survival (RFS) outcomes to lobectomy, showing low heterogeneity. On the other hand, wedge resection was associated with inferior OS and RFS outcomes compared to lobectomy. However, subgroup analysis based on age showed comparable outcomes between wedge resection and lobectomy for patients aged over 75 years.

Lung cancer is a multifaceted disease encompassing different subtypes, each exhibiting distinct clinical and radiological characteristics^1^. This heterogeneity necessitates a customized approach to the management of lung cancer, taking into account the specific features of solid-dominant and ground-glass-dominant subtypes^2^.

Historically, lobectomy has been the gold standard surgical procedure for the resection of early-stage lung cancer, as established by the landmark Lung Cancer Study Group randomized trial conducted in 1995^3^. The trial compared lobectomy with limited resection for stage IA lung cancer. However, with the advent of low-dose computed tomography (CT) screening, there has been an increase in the detection of early-stage lung cancer, particularly small and peripheral lung cancers^4^.

The increased identification of early-stage lung cancer and the diverse patient population presenting with these tumors have influenced the adoption of sublobar resection as an alternative surgical approach^5^. Sublobar resection, including segmentectomy and wedge resection, aims to preserve lung function while achieving adequate oncologic control^6,7^. The feasibility of sublobar resection has been supported by studies demonstrating that ground-glass opacity (GGO)-dominant tumors, characterized by a wide area of GGO on CT scans, are associated with a good prognosis and can be effectively treated with this approach^8,9^.

However, solid-dominant lung cancer is characterized by its aggressive nature and higher likelihood of lymph node involvement^10^. The optimal surgical approach for managing this type of tumor is currently a subject of intense debate^11^. Some studies have indicated that the recurrence-free survival (RFS) and overall survival (OS) rates were lower after sublobar resection than after lobectomy for solid-dominant clinical stage IA lung cancer^12,13^. On the contrary, several recent studies have presented different results, suggesting that sublobar resection could have similar long-term effects to lobectomy for radiologically solid-dominant stage IA lung cancer^14,15^.

This meta-analysis aimed to address the knowledge gap by evaluating whether sublobar resection (including segmentectomy and wedge resection) provides comparable oncologic outcomes to lobectomy in stage IA lung cancer patients with a solid-dominant appearance. By synthesizing and analyzing the existing literature, this study will contribute to the understanding of the optimal surgical management for solid-dominant stage IA lung cancer.

## Methods

This meta-analysis was conducted following the guidelines of the PRISMA (Preferred Reporting Items for Systematic Reviews and Meta-Analyses) (Supplemental Digital Content 1, http://links.lww.com/JS9/B324)^16^ and AMSTAR (Assessing the methodological quality of systematic reviews) (Supplemental Digital Content 2, http://links.lww.com/JS9/B325)^17^. Furthermore, the study was registered in the PROSPERO database (International Prospective Register of Systematic Reviews) with registration number CRD42023430350, which is available at https://www.crd.york.ac.uk/prospero/display_record.php?ID=CRD42023430350.

### Literature search

A systematic literature search was conducted from the start of available records to 15 May 2023 using the following databases: PubMed, Web of Science, Cochrane Library, Embase, and Scopus. The aim was to compare sublobar resection (including segmentectomy and wedge resection) with lobectomy for solid-dominant stage IA lung cancer in terms of OS and RFS. The search terms used were as follows (example using PubMed): ((((((pulmonary[Title/Abstract]) OR (lung[Title/Abstract])) AND ((((cancer*[Title/Abstract]) OR (carcinoma*[Title/Abstract])) OR (adenocarcinoma*[Title/Abstract])) OR (neoplasm*[Title/Abstract]))) AND (((((solid-dominant[Title/Abstract]) OR (pure solid[Title/Abstract])) OR (solid*[Title/Abstract])) OR (consolidation*[Title/Abstract])) OR (CTR[Title/Abstract]))) AND ((((((segmentectomy[Title/Abstract]) OR (segmental resection[Title/Abstract])) OR (wedge resection[Title/Abstract])) OR (sublobectomy[Title/Abstract])) OR (sublobar resection[Title/Abstract])) OR (limited resection[Title/Abstract]))) AND (((lobectomy[Title/Abstract]) OR (lobar resection[Title/Abstract])) OR (anatomical resection[Title/Abstract]))) AND (((survival*) OR (prognos*)) OR (outcome*)). After removing duplicates, two independent reviewers (L.H.H. and P.Z.Y.) screened the titles and abstracts of the initial search findings to identify candidate studies for inclusion. Candidate studies were retrieved and evaluated based on the inclusion and exclusion criteria. The final decision for inclusion was made based on consensus between the two reviewers.

### Study selection

The inclusion criteria were as follows: (1) solid-dominant (consolidation-to-tumor ratio (CTR) ≥50%) clinical stage IA lung cancer, (2) comparative studies within the same study comparing sublobar resection (including segmentectomy and wedge resection) with lobectomy, including randomized controlled trial (RCT) and retrospective cohort studies (RCS), (3) reporting at least one outcome of interest, including OS or RFS, and (4) publication in English language. The exclusion criteria were as follows: (1) studies with overlapping study populations, (2) review articles, editorials, letters, abstracts, non-original studies, and animal studies, and (3) studies that did not report the outcomes of interest.

### Data extraction and study quality assessment

Two review authors independently extracted relevant data from the included studies, including author, year, study type, country, study period, center, sample, surgical procedure, operation timing, age, and outcomes of interest (OS and RFS). Hazard ratio (HR) was used as a measure for comparison and was extracted from the text or the published Kaplan–Meier curves of survival estimates. HR with its 95% confidence interval (CI) was calculated following the guidelines of Tierney *et al*.^18^. The quality of RCS was assessed using the Newcastle–Ottawa Quality Assessment Scale (NOS)^19^, while the risk of bias in RCT was evaluated using the Cochrane Collaboration’s Risk of Bias Tool^20^.

### Statistical analyses

The statistical analyses were performed using the R language. The meta-analysis was conducted to combine the results from individual studies and obtain summary estimates of the effect size. For the outcome measures of interest, such as OS and RFS, HRs and their corresponding 95% CIs were used as the effect size measures. To assess the heterogeneity among the included studies, the *I*-squared (*I*^2^) statistic was calculated. *I*^2^ values greater than 50% indicate substantial heterogeneity. In the presence of significant heterogeneity, a random-effects model was employed to estimate the pooled effect size. Conversely, if the heterogeneity was low (*I*^2^ ≤50%), a fixed-effects model was used. Subgroup analyses were conducted to explore potential sources of heterogeneity and assess the impact of specific factors, such as tumor size, CTR, surgical approaches, and different age groups. Stratified analyses based on these factors were performed, and subgroup differences were assessed using statistical tests.

Publication bias was evaluated using Egger’s test, Begg’s test, and contour-enhanced funnel plots with the trim-and-fill method. If evidence of publication bias was detected, the trim-and-fill method was employed to re-estimate the pooled results, assessing the stability of the findings after adjusting for potential publication bias. Sensitivity analyses were performed to assess the robustness of the results. This involved systematically excluding one study at a time and recalculating the pooled effect size to examine the influence of individual studies on the overall results. All statistical tests were two-sided, and a *P*-value less than 0.05 was considered statistically significant.

## Results

### Characteristics of the included studies

**Figure 1:**
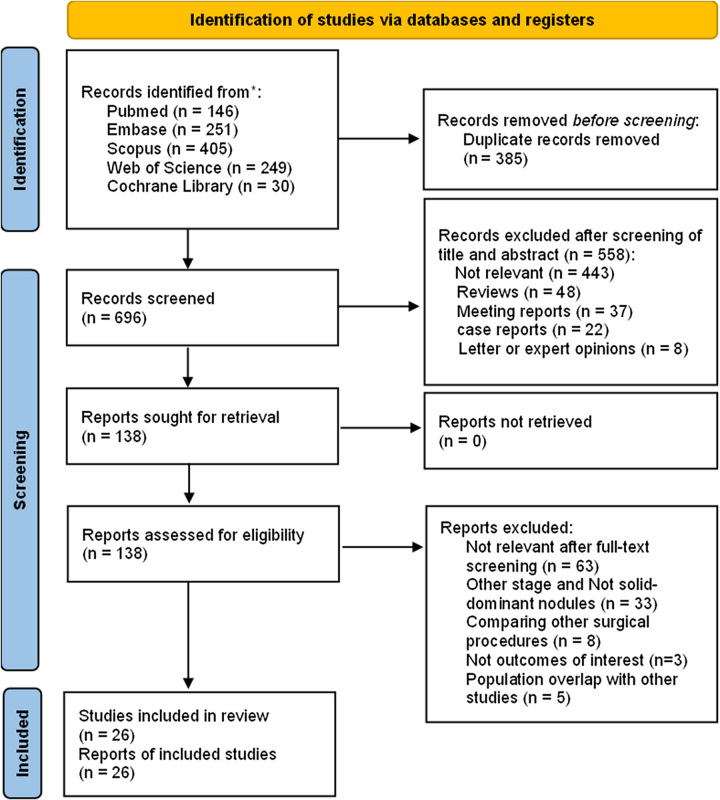
Flowchart of study selection process.

As was shown in Figure 1, a total of 1081 publications were identified in the initial search. After removing 385 duplications, 696 articles underwent screening based on title and abstract. Subsequently, 558 studies were excluded, leaving 138 articles for detailed evaluation of the full text. It should be noted that five studies were excluded as their subjects were already included in the selected studies. Ultimately, 26 articles were included in this study, consisting of one RCT and the remaining being RCS. Among these, 16 studies were conducted in Japan, 6 in China, 2 in Korea, 1 in America, and 1 in Switzerland. A total of 12 667 patients were included in this study, with 3488 patients in the sublobar resection group and 9179 patients in the lobectomy group. A total of 392 (out of 2763) patients were mentioned to receive adjuvant therapy, with 91 (out of 1052) in the sublobar resection group and 228 (out of 1638) in the lobectomy group. Among the patients in the sublobar resection group, 1052 (out of 1129) and in the lobectomy group, 1582 (out of 1748) received video-assisted thoracic surgery, respectively. Table 1 presented the characteristics of each study.

**Table 1:** List of included studies.

Author	Year	Country	Study design	Study period	Nodule size	Density of the nodule	Number of patients	Number of patients receiving adjuvant therapy	Number of patients undergoing VATS
Altorki *et al*.	2021^21^	Japan	RCS PSM	1993–2011	≤3 cm	Pure solid	Seg/WR: 16/37 Lob: 294	—	—
Darras *et al*.	2021^22^	Switzerland	RCS	2014–2019	<2 cm	Pure solid	Seg: 84 Lob: 84	Seg: 11 Lob: 14	Seg: 84 Lob: 84
Hattori *et al*.	2016^40^	Japan	RCS	2008–2013	2–3 cm	Solid-dominant	Seg: 31 Lob: 123	Seg: 1 Lob: 36	—
Hattori *et al*.	2017^23^	Japan	RCS	2008–2014	≤2 cm	Solid-dominant	Seg: 83 Lob: 270	Seg/Lob:73	—
Hattori *et al*.	2017^24^	Japan	RCS	2008–2013	≤3 cm	Pure solid	SLR: 35 Lob: 165	—	—
Hattori *et al*.	2022^25^	Japan	RCS	2008–2018	2–3 cm	Solid-dominant	Seg: 46 Lob: 169	Seg: 4 Lob: 61	Seg: 46 Lob: 169
Huang *et al*.	2020^39^	China	RCS	2006–2016	≤3 cm	Solid-dominant	SLR: 55 Lob: 348	—	—
Inoue *et al*.	2010^26^	Japan	RCS	1992–2007	≤2 cm	Pure solid	SLR: 28 Lob: 90	—	—
Jeon *et al*.	2014^12^	Korea	RCS	2000–2010	≤3 cm	Pure solid	SLR: 31 Lob: 133	SLR: 8 Lob: 22	—
Kamigaichi *et al*.	2020^27^	Japan	RCS PSM	2007–2017	2.1–3 cm	Solid-dominant	Seg: 37 Lob: 37	—	Seg: 37 Lob: 37
Kamigaichi *et al*.	2020^28^	Japan	RCS PSM	2010–2016	≤2 cm	Solid-dominant	Seg: 115 Lob: 115	Seg: 18 Lob: 19	Seg: 115 Lob: 115
Kamigaichi *et al*.	2016^29^	Japan	RCS	2010–2019	≤2 cm	Solid-dominant	WR: 193 Lob: 530	WR: 5 Lob: 9	—
Koike *et al*.	2016^30^	Japan	RCS PSM	1998–2009	≤2 cm	Pure solid	Seg: 87 Lob: 87	—	—
Li *et al*.	2023^31^	China	RCS PSM	2010–2019	≤2 cm	Pure solid	Seg: 74 Lob: 74	—	—
Mimae *et al*.	2020^32^	Japan	RCS	2010–2016	≤2 cm	Solid-dominant	WR: 28 Lob: 21	—	—
Nishio *et al*.	2016^33^	Japan	RCS PSM	1992–2009	≤2 cm	Solid-dominant	Seg: 59 Lob: 59	—	Seg: 59 Lob: 59
Phillips *et al*.	2020^34^	America	RCS	2000–2017	≤3 cm	Solid-dominant	SLR: 161 Lob: 196	—	—
Saji *et al*.	2022^15^	Japan	RCT	2009–2014	≤2 cm	Solid-dominant	Seg: 552 Lob: 554	Seg: 44 Lob: 67	Seg: 495 Lob: 491
Soh *et al*.	2022^35^	Japan	RCS PSM	—	≤3 cm	Solid-dominant	Seg/WR: 657/ 712 Lob: 4323	—	—
Su *et al*.	2018^41^	China	RCS	2010–2013	≤3 cm	Solid-dominant	SLR: 10 Lob: 56	—	—
Su *et al*.	2020^36^	China	RCS	2009–2013	≤2 cm	Pure solid	Seg/WR: 54/85 Lob: 633	—	Seg/WR: 47/72 Lob: 530
Suh *et al*.	2018^42^	Korea	RCS	2006–2016	≤3 cm	Pure solid	WR: 36 Lob: 275	—	—
Sun *et al*.	2020^43^	China	RCS	2012–2015	≤3 cm	Solid-dominant	SLR: 18 Lob: 132	—	—
Tsutani *et al*.	2014^44^	Japan	RCS	2005–2010	≤3 cm	Solid-dominant	Seg: 41 Lob: 286	—	—
Tsutani *et al*.	2021^37^	Japan	RCS	2006–2010	≤3 cm	Solid-dominant	SLR: 26 Lob: 28	—	—
Wu *et al*.	2023^38^	China	RCS	2016–2018	≤2 cm	Solid-dominant	Seg/WR: 16/81 Lob: 97	—	Seg/WR: 16/81 Lob: 97

Lob, lobectomy; PSM, propensity score matching; RCS, retrospective cohort studies; RCT, randomized controlled trial; Seg, segmentectomy; SLR, sublobar resection; VATS, video-assisted thoracic surgery; WR, wedge resection.

### Quality assessment of the studies

**Figure 2:**
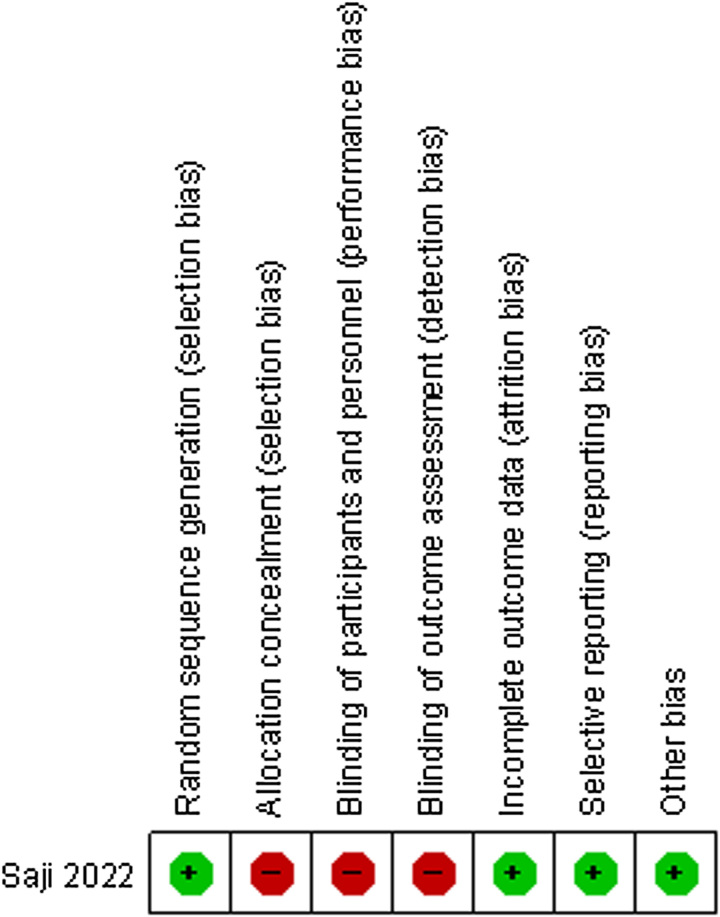
Risk of bias assessment of RCT.

The quality of RCS was assessed using the NOS, with scores ranging from 5 to 8 (Table 2). RCT was evaluated using the Cochrane Collaboration’s Risk of Bias Tool. The results were shown in Figure 2.

**Table 2:** Quality score of retrospective cohort studies based on the Newcastle–Ottawa quality assessment scale.

	Selection			Comparability			Outcome			
Study	REC	SNEC	AE	DO	SC	AF	AO	FU	AFU	Total stars
Altorki *et al*., 2014^21^	1	1	1	1	1	1	1	1		8
Darras *et al*., 2021^22^	1	1	1	1	1		1			6
Hattori *et al*., 2016^40^	1	1	1	1	1	1	1			7
Hattori *et al*., 2017^23^	1	1	1	1			1	1		6
Hattori *et al*., 2017^24^	1	1	1	1	1		1			6
Hattori *et al*., 2022^25^	1	1	1	1	1		1	1		7
Huang *et al*., 2020^39^	1	1	1	1			1	1		6
Inoue *et al*., 2010^26^	1	1	1	1			1	1		6
Jeon *et al*., 2014^12^	1	1	1	1	1		1	1		7
Kamigaichi, 2020^27^	1	1	1	1	1	1	1	1		8
Kamigaichi, 2020^28^	1	1	1	1	1	1	1	1		8
Kamigaichi *et al*., 2023^29^	1	1	1	1	1		1	1		7
Koike *et al*., 2016^30^	1	1	1	1	1	1	1	1		8
Li *et al*., 2023^31^	1	1	1	1	1	1	1	1		8
Mimae *et al*., 2020^32^		1	1	1	1		1			5
Nishio *et al*., 2016^33^	1	1	1	1	1	1	1	1		8
Phillips *et al*., 2020^34^		1	1	1	1		1	1		6
Soh *et al*., 2022^35^	1	1	1	1	1	1	1	1		8
Su *et al*., 2018^41^	1	1	1	1	1		1	1		7
Su *et al*., 2020^36^		1	1	1	1		1	1		6
Suh *et al*., 2018^42^	1	1	1	1	1		1	1		7
Sun *et al*., 2020^43^	1	1	1	1			1	1		6
Tsutani *et al*., 2021^37^	1	1	1	1	1		1	1		7
Tsutani *et al*., 2021	1	1	1	1			1	1		6
Wu *et al*., 2023^38^		1	1	1	1	1	1	1		7

AE, ascertainment of exposure; AF, study controls for other important factors; AFU, adequacy of follow-up of cohort (≥80%); AO, assessment of outcome; DO, demonstration that outcome of interest was not present at start of study; FU, follow-up long enough for outcomes to occur; REC, representativeness of the cohort; SC, study controls most important factors; SNEC, selection of the none posed cohort.

### Pooled results of all studies comparing sublobar resection and lobectomy

**Figure 3:**
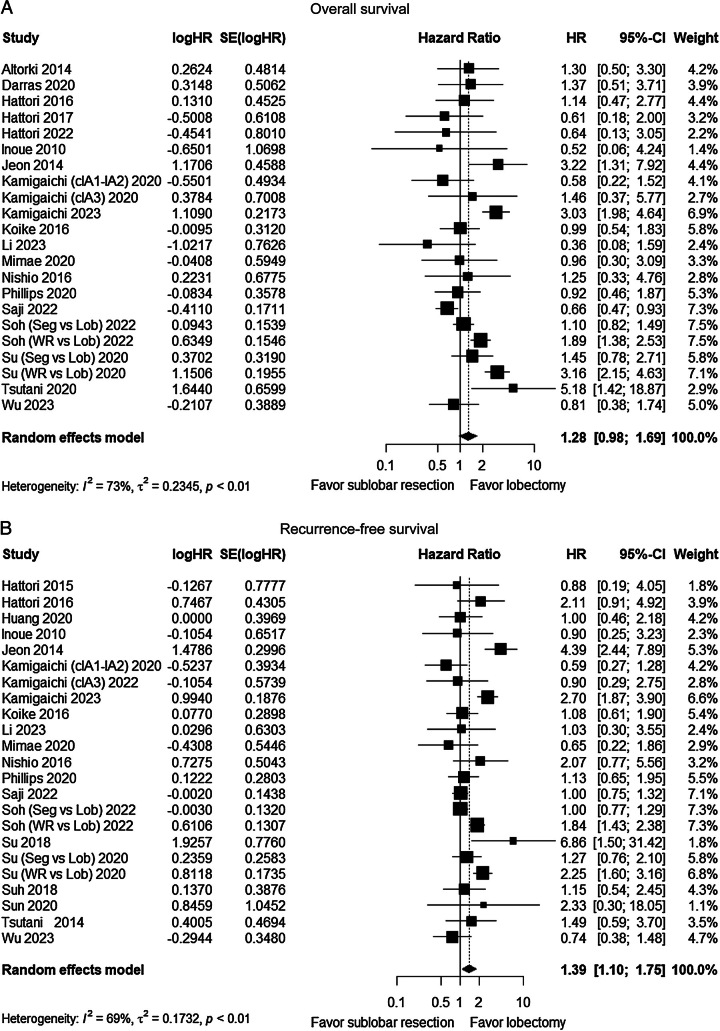
Forest plot of survival comparing sublobar resection and lobectomy. (A) Overall survival (OS). (B) Recurrence-free survival (RFS). Lob, lobectomy; Seg, segmentectomy; WR, wedge resection.

A total of 20 studies compared OS between the sublobar resection group and the lobectomy group^12,15,21–38^, and 21 studies compared RFS^12,15,23,26–36,38–44^, respectively. The pooled results showed no statistically significant difference in OS between the two groups (HR=1.28, 95% CI: 0.98–1.69, *P*=0.07) (Fig. 3A), but there was significant heterogeneity among the studies (*I*^2^=73%, *P*<0.01). However, lobectomy was associated with better RFS (HR=1.39, 95% CI: 1.10–1.75, *P*<0.01) (Fig. 3B), with significant heterogeneity among the studies (*I*^2^=69%, *P*<0.01).

### Pooled results of studies comparing sublobar resection and lobectomy in different populations

The effectiveness of sublobar resection versus lobectomy in different patient populations was compared, and the results were presented in Table 3.

**Table 3:** Pooled results of studies comparing sublobar resection and lobectomy in different populations.

Tumor size	CTR	Surgical approach	HR (OS)	*I*^2^ (OS)	HR (RFS)	*I*^2^ (RFS)
≤3 cm	0.5–1	Seg vs. Lob	0.93 (0.78–1.12)	10%	1.06 (0.91–1.24)	0%
≤3 cm	0.5–1	WR vs. Lob	1.98 (1.30–3.02)	64%	1.69 (1.11–2.38)	66%
≤2 cm	0.5–1	SLR vs. Lob	1.34 (0.95–1.89)	82%	1.29 (1.01–1.65)	75%
2–3 cm	0.5–1	SLR vs. Lob	2.74 (1.01–7.44)	67%	2.17 (1.45–3.24)	35%
≤3 cm	≥0.5, <1	SLR vs. Lob	1.81 (0.94–3.49)	77%	1.85 (1.45–2.38)	35%
≤3 cm	1	SLR vs. Lob	1.44 (1.08–1.91)	77%	1.60 (1.27–2.01)	71%
≤2 cm	0.5–1	Seg vs. Lob	1.02 (0.86–1.21)	39%	1.06 (0.92–1.23)	3%
2–3 cm	0.5–1	Seg vs. Lob	1.89 (0.61–5.87)	66%	1.55 (0.65–3.67)	54%
≤2 cm	0.5–1	WR vs. Lob	1.55 (0.86–2.81)	91%	1.69 (1.12–2.55)	70%
2–3 cm	0.5–1	WR vs. Lob	7.40 (2.95–18.58)	–	2.57 (1.24–5.32)	–

CTR, consolidation-to-tumor ratio; HR, hazard ratio; Lob, lobectomy; OS, overall survival; RFS, recurrence-free survival; Seg, segmentectomy; SLR, sublobar resection; WR, wedge resection.

For lung cancers with a size of ≤2 cm, there was no statistically significant difference in OS between sublobar resection and lobectomy (HR=1.34, 95% CI: 0.95–1.89, *I*^2^=82%). However, it is important to note that sublobar resection was associated with a significantly lower RFS compared to lobectomy (HR=1.29, 95% CI: 1.01–1.65, *I*^2^=75%). In the case of 2–3 cm lung cancers, both OS and RFS in the sublobar resection group were significantly lower than in the lobectomy group (OS: HR=2.74, 95% CI: 1.01–7.44, *I*^2^=67%; RFS: HR=2.17, 95% CI: 1.45–3.24, *I*^2^=35%).

For tumors with 0.5≤CTR<1, there was no statistically significant difference in OS between sublobar resection and lobectomy (HR=1.81, 95% CI: 0.94–3.49, *I*^2^=77%), but sublobar resection was associated with a significantly lower RFS compared to lobectomy (HR=1.85, 95% CI: 1.45–2.38, *I*^2^=35%). When dealing with CTR=1 lung cancers, both OS and RFS in the sublobar resection group were significantly lower than in the lobectomy group (OS: HR=1.44, 95% CI: 1.08–1.91, *I*^2^=77%; RFS: HR=1.60, 95% CI: 1.27–2.01, *I*^2^=71%).

Furthermore, subgroup analyses based on surgical methods were conducted. Compared to lobectomy, segmentectomy provided patients with similar OS and RFS (OS: HR=0.93, 95% CI: 0.78–1.12, *I*^2^=10%; RFS: HR=1.06, 95% CI: 0.91–1.24, *I*^2^=0%). However, when compared to lobectomy, wedge resection had a detrimental effect on patient prognosis (OS: HR=1.98, 95% CI: 1.30–3.02, *I*^2^=64%; RFS: HR=1.63, 95% CI: 1.11–2.38, *I*^2^=66%).

Further analysis revealed that segmentectomy did not compromise the prognosis of patients with tumors ≤2 cm (OS: HR=1.02, 95% CI: 0.86–1.21, *I*^2^=39%; RFS: HR=1.06, 95% CI: 0.92–1.23, *I*^2^=3%) and patients with 2–3 cm tumors (OS: HR=1.89, 95% CI: 0.61–5.87, *I*^2^=66%; RFS: HR=1.55, 95% CI: 0.65–3.67, *I*^2^=54%). On the other hand, wedge resection did not significantly affect OS in patients with tumors ≤2 cm (HR=1.55, 95% CI: 0.86–2.81, *I*^2^=91%), but it did decrease RFS in patients with tumors ≤2 cm (HR=1.69, 95% CI: 1.12–2.55, *I*^2^=70%) and survival in patients with 2–3 cm tumors (OS: HR=7.40, 95% CI: 2.95–18.58; RFS: HR=2.57, 95% CI: 1.24–5.32).

To further ascertain the role of wedge resection in stage IA solid-dominant lung cancer, the studies were categorized into two groups: studies involving patients across various age groups and those involving patients aged 75 years or older. For studies including patients of different age groups, wedge resection showed worse OS and RFS (OS: HR=2.43, 95% CI: 1.98–2.98, *I*^2^=48%; RFS: HR=2.06, 95% CI: 1.73–2.46, *I*^2^=44%) (Table 4). However, for patients aged over 75 years, wedge resection provided comparable OS and RFS (OS: HR=0.81, 95% CI: 0.39–1.69, *I*^2^=0%; RFS: HR=0.85, 95% CI: 0.49–1.49, *I*^2^=0%) (Table 4).

**Table 4:** Subgroup analysis of survival comparing wedge resection and lobectomy based on patients’ age.

Tumor size	CTR	Surgical approach	Age groups	HR (OS)	*I*^2^ (OS)	HR (RFS)	*I*^2^ (RFS)
≤3 cm	0.5–1	WR vs. Lob	Various age groups	2.43 (1.98–2.98)	48%	2.06 (1.73–2.46)	44%
≤3 cm	0.5–1	WR vs. Lob	≥75 years	0.81 (0.39–1.69)	0%	0.85 (0.49–1.49)	0%

CTR, consolidation-to-tumor ratio; HR, hazard ratio; Lob, lobectomy; OS, overall survival; RFS, recurrence-free survival; WR, wedge resection.

### Publication bias

Initially, Egger’s test and Begg’s test were employed to assess potential publication bias. As was shown in Table 5, the results of Egger’s test and Begg’s test indicated no significant publication bias in the comparisons of sublobar resection versus lobectomy for both OS and RFS (OS: Egger’s test: *P*=0.4196, Begg’s test: *P*=0.6118; RFS: Egger’s test: *P*=0.8019, Begg’s test: *P*=0.6345).

**Table 5:** Results of Egger’s test and Begg’s test.

Comparison	Outcome	*P* of Egger’s test	*P* of Begg’s test
Sublobar resection vs. lobectomy	OS	0.4196	0.6118
	RFS	0.8019	0.6345

OS, overall survival; RFS, recurrence-free survival.

Contour-enhanced funnel plot with trim-and-fill method has been proposed as a valuable assessment method for investigating the potential causes of funnel plot asymmetry^45^. Contour lines with statistical significance of 0.01, 0.05, and 0.1 are depicted in the funnel plot. The inclusion of new supplementary studies within the non-statistically significant interval suggests that the asymmetry in the funnel plot could be attributed to publication bias. Therefore, contour-enhanced funnel plots can be utilized to identify potential publication bias and the trim-and-fill method can be employed to adjust the results.

**Figure 4:**
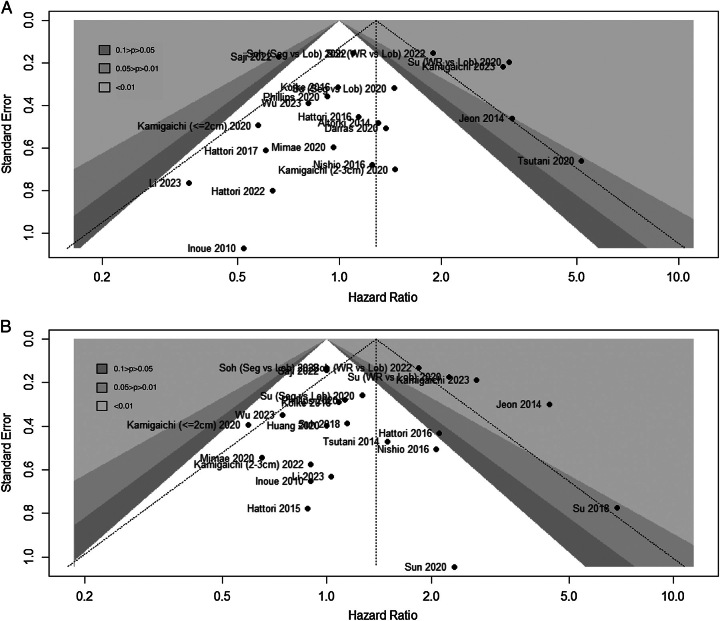
Contour-enhanced funnel plot of survival between different surgical procedures. (A) Overall survival (OS) between sublobar resection and lobectomy. (B) Recurrence-free survival (RFS) between sublobar resection and lobectomy. Lob, lobectomy; Seg, segmentectomy; WR, wedge resection.

**Figure 5:**
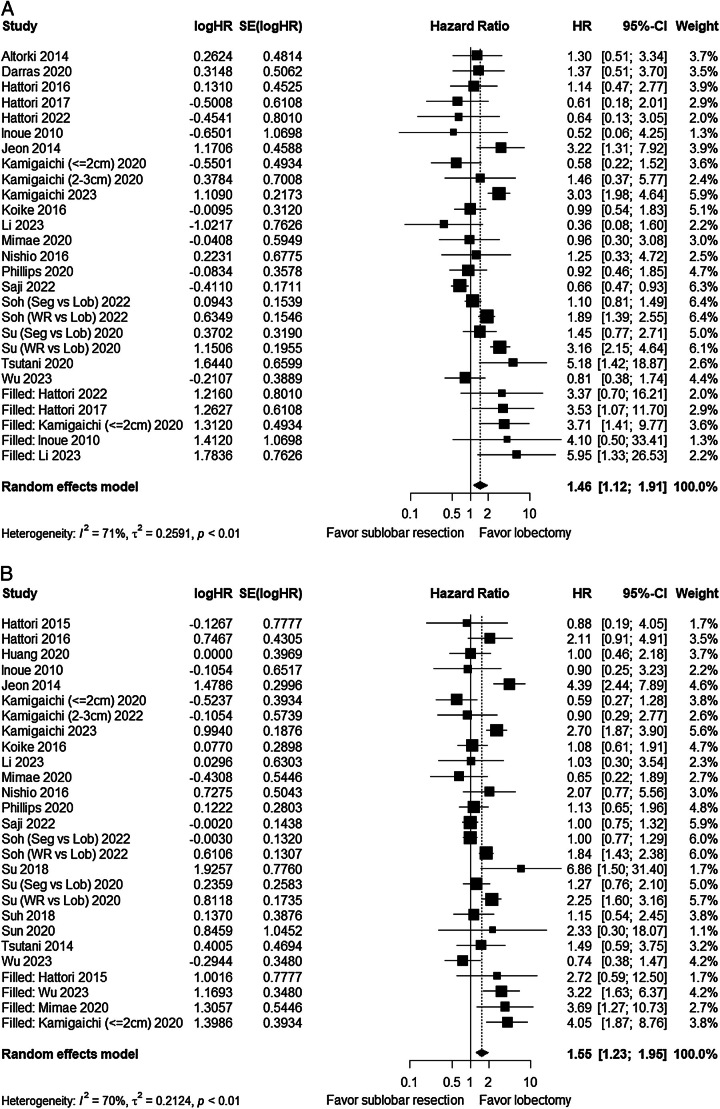
Adjusted result of survival between different surgical procedures using trim-and-fill method. (A) Overall survival (OS) between sublobar resection and lobectomy. (B) Recurrence-free survival (RFS) between sublobar resection and lobectomy. Lob, lobectomy; Seg, segmentectomy; WR, wedge resection.

For the pooled OS and RFS results of all studies, there were missing studies in the non-significant area (Fig. 4A, B), indicating a certain degree of publication bias. After adjustment, the results showed a statistically significant difference in OS between the two groups (HR=1.46, 95% CI: 1.12–1.91, *I*^2^=71%, *P*<0.01) (Fig. 5A), while the trend for RFS remained unchanged (HR=1.55, 95% CI: 1.23–1.96, *I*^2^=70%, *P*<0.01) (Fig. 5B).

### Sensitivity analysis

**Figure 6:**
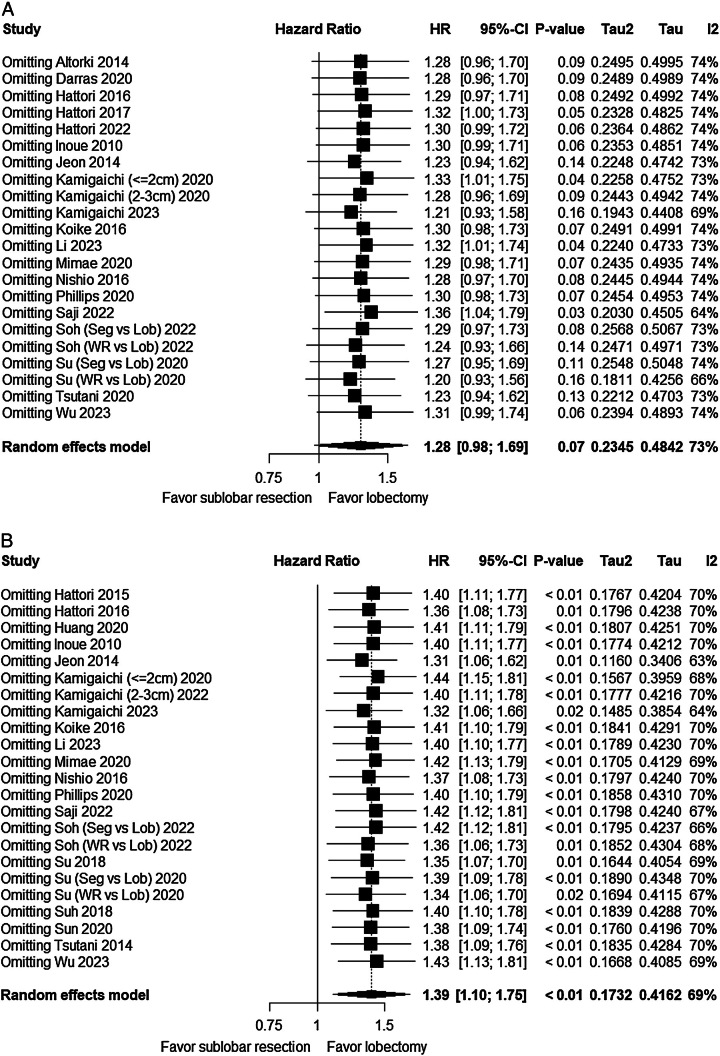
Sensitivity analysis result of survival between different surgical procedures. (A) Overall survival (OS) between sublobar resection and lobectomy. (B) Recurrence-free survival (RFS) between sublobar resection and lobectomy. Lob, lobectomy; Seg, segmentectomy; WR, wedge resection.

To assess the influence of individual studies on the results, sensitivity analysis was conducted by removing each study individually. For the pooled results of all studies, sensitivity analysis indicated instability in OS (*P*: 0.03–0.16) (Fig. 6A), while RFS remained stable (*P*: <0.01–0.02) (Fig. 6B).

## Discussion

In 2019, two meta-analyses were published comparing sublobar resection to lobectomy for solid-dominant stage IA lung cancer^13,14^.However, these meta-analyses yielded conflicting conclusions and included a limited number of primary studies. The results of a RCT in 2022 (JCOG0802/WJOG4607L study) demonstrated that segmentectomy was non-inferior and superior to lobectomy in terms of OS for solid-dominant stage IA non-small cell lung cancer with tumor diameter ≤2 cm^15^. Therefore, the study recommended segmentectomy as the standard surgical procedure for this patient population instead of lobectomy. However, the trial has limitations including its non-blinded design, which may introduce inevitable bias, and the need for further clarification on the reasons why segmentectomy is superior to lobectomy. Thus, further investigation of the role of sublobar resection in solid-dominant stage IA lung cancer is necessary.

In this meta-analysis, we aimed to evaluate the oncological outcomes of sublobar resection, specifically segmentectomy and wedge resection, compared to lobectomy in patients with solid-dominant stage IA lung cancer. Our findings indicated that when sublobar resection was analyzed as a whole, the pooled results for OS and RFS showed significant heterogeneity. Therefore, we proceeded to conduct subgroup analyses to explore the potential impact of different factors (tumor size, CTR, and surgical approach) on the results.

Currently, most studies suggest that tumor size and CTR are pivotal factors influencing both the prognosis of early-stage lung cancer and the decision-making process for surgical interventions^2,46^. Our analysis considered these factors separately to better understand their individual effects on patient outcomes. Our findings consistently highlighted that sublobar resection, particularly wedge resection, had a detrimental impact on the prognosis of patients across different tumor sizes (≤2 cm and 2–3 cm) and CTR categories (0.5≤CTR<1 and CTR=1). Notably, this was especially evident in terms of increasing the risk of postoperative recurrence. Despite our efforts to stratify the analysis based on tumor size and CTR, it was essential to acknowledge that the combined results still exhibited substantial heterogeneity. This suggested that factors beyond tumor size and CTR may contribute to the variations in patient outcomes, warranting further investigation. Therefore, we proceeded to analyze segmentectomy and wedge resection separately to explore their individual effects on patient outcomes compared with lobectomy.

Segmentectomy has been proposed as an alternative to lobectomy for early-stage lung cancer, particularly in patients with limited pulmonary function or comorbidities that make lobectomy challenging^47^. Our meta-analysis revealed that segmentectomy yielded comparable oncological outcomes to lobectomy in terms of OS and RFS with low heterogeneity. This finding supported the notion that segmentectomy can be a reasonable option for solid-dominant stage IA lung cancer patients, providing them with a less invasive surgical approach while maintaining comparable survival outcomes. To reinforce our findings, we performed additional subgroup analyses within the segmentectomy versus lobectomy studies, specifically categorizing them by tumor size (≤2 cm and 2–3 cm). In both subgroups, segmentectomy did not compromise OS or RFS compared to lobectomy, which strengthened the evidence supporting the comparable efficacy of segmentectomy in this patient population.

Solid-dominant tumors are known to exhibit aggressive biological behavior, often involving a higher degree of invasiveness and lymph node involvement^10^. While segmentectomy may seem less intuitive due to the potential aggressiveness of solid-dominant tumors, studies have demonstrated that segmentectomy can still address these factors adequately^48,49^. The possible reason is that segmentectomy allows for sufficient resection margins and thorough evaluation of lymph nodes, ensuring comprehensive management of the oncological aspects associated with solid-dominant stage IA lung cancer^50^.

In addition, segmentectomy offers several advantages over lobectomy. Firstly, by removing a specific lung segment, segmentectomy minimizes the loss of healthy lung tissue. This is particularly important in patients with compromised lung function, such as those with pre-existing respiratory conditions or limited pulmonary reserve. Preservation of lung function can lead to improved postoperative respiratory outcomes, reduced risk of complications, and better overall quality of life for patients undergoing segmentectomy^51,52^. Moreover, segmentectomy preserves a greater amount of lung parenchyma, which may contribute to more extensive treatment options. This includes addressing the recurrence of primary lung cancer and second primary lung cancer, as well as potentially providing treatment possibilities for other malignancies and life-threatening diseases that may be present^15^.

In addition to segmentectomy, wedge resection has also been considered as a surgical option for solid-dominant stage IA lung cancer patients. Our meta-analysis also aimed to evaluate the oncological outcomes of wedge resection compared to lobectomy in this patient population. The pooled results suggested that wedge resection may compromise OS and RFS compared to lobectomy. In further analysis, while wedge resection may not compromise the OS of patients with tumors ≤2 cm, it did increase the risk of recurrence. Additionally, wedge resection was associated with lower survival in the 2–3 cm population. Wedge resection may result in poorer prognosis for solid-dominant stage IA lung cancer patients due to several factors. Firstly, wedge resection involves a more limited resection of the lung tissue, potentially leaving behind microscopic tumor cells. This may increase the risk of local recurrence and compromise OS. Secondly, wedge resection often does not include a thorough evaluation of lymph nodes, which can affect accurate staging and subsequent treatment decisions. Inadequate lymph node assessment may lead to undertreatment or missed opportunities for adjuvant therapy.

To investigate which subset of patients might benefit from wedge resection, we conducted a subgroup analysis based on patient age, categorizing the studies into those including patients from various age groups and those involving patients aged 75 years or older. The results of this analysis indicated that wedge resection may be a feasible option for older patients, as it demonstrated similar OS and RFS outcomes compared to lobectomy. These findings suggested that advanced age alone should not preclude the consideration of wedge resection in elderly patients with solid-dominant stage IA lung cancer. Several key factors support this conclusion.

Firstly, wedge resection results in shorter operative times, reduced blood loss, shorter hospitalization durations, and fewer postoperative complications^53^. Secondly, due to the physiological frailty and reduced lung capacity in elderly patients, they often die from non-neoplastic causes, including respiratory disease and cerebrovascular disease, during the course of tumor treatment, which happens to be the disadvantage of lobectomy^15,54,55^. Moreover, wedge resection may have slightly inferior prognosis compared to other surgical methods in early-stage lung cancer patients. However, for tumors with specific characteristics, such as a favorable prognosis and lower pathological invasiveness, particularly those with GGO components, wedge resection can still provide sufficient cancer control^29^. Therefore, in cases that meet the criteria for low-grade tumors, wedge resection can offer appropriate cancer control for elderly patients.

Based on the above discussion, it is evident that segmentectomy and wedge resection exhibit varying effects compared to lobectomy in the management of solid-dominant stage IA lung cancer. Segmentectomy emerges as a surgical approach that is associated with minimal impairment to patient prognosis. On the other hand, wedge resection appears to be accompanied by a compromised prognosis. It is noteworthy, however, that wedge resection may still have a role to play in select cases, particularly among elderly patients. Therefore, careful consideration of the patient’s age and individual circumstances is crucial when determining the appropriate surgical intervention for solid-dominant stage IA lung cancer.

It is essential to acknowledge the limitations of our meta-analysis. Firstly, the included studies exhibited inherent variations in patient characteristics, surgical techniques, and follow-up durations, which likely contributed to the observed heterogeneity. Secondly, the majority of the studies were retrospective in nature, raising the potential for biases that may have influenced the results. Moreover, certain important factors, including postoperative complications, quality of life, and functional outcomes, were not thoroughly addressed in this analysis. Future studies should aim to address these limitations and provide a more comprehensive evaluation of surgical modalities for solid-dominant stage IA lung cancer patients, considering these factors and potential biases.

## Conclusion

In conclusion, our meta-analysis suggests that segmentectomy is a reasonable choice for solid-dominant stage IA lung cancer patients, offering comparable oncological outcomes to lobectomy. However, the use of wedge resection requires careful consideration, particularly in patients other than the elderly. The findings of this study contribute to the ongoing discussion on the optimal surgical approach for early-stage lung cancer and provide valuable insights for clinical decision-making. Further well-designed studies are warranted to confirm and expand upon these findings, taking into account various patient factors and long-term outcomes.

## Ethical approval

This study did not require ethical approval.

## Consent

Data used for meta-analysis were extracted from previously published papers.

## Sources of funding

This work is supported by the 1.3.5 Project for Disciplines of Excellence (ZYJC18009), West China Hospital, Sichuan University, to Dr Jiandong Mei.

## Author contribution

Study concept and design: H.L. and J.M.; acquisition of data: H.L., Z.P., K.Z., L.L., J.C., and Z.H.; analysis and interpretation of data: H.L., Z.P., J.M., and L.C.; drafting of the manuscript: H.L., Z.P., and J.M.; critical revision of the manuscript for important intellectual content: all authors; study supervision: J.M. and L.C.

## Conflicts of interest disclosure

The authors declare no potential conflicts of interest.

## Research registration unique identifying number (UIN)

1. Name of the registry: PROSPERO.
2. Unique identifying number or registration ID: CRD42023430350.
3. Hyperlink to your specific registration (must be publicly accessible and will be checked): https://www.crd.york.ac.uk/prospero/display_record.php?ID=CRD42023430350.

## Guarantor

Jiandong Mei.

## Date availability statement

This is a summary design study. Data used for meta-analysis were extracted from previously published papers.

## Provenance and peer review

Not commissioned, externally peer-reviewed.

## Supplementary Material
